# Induction of Avian β-Defensin 2 Is Possibly Mediated by the p38 MAPK Signal Pathway in Chicken Embryo Fibroblasts After Newcastle Disease Virus Infection

**DOI:** 10.3389/fmicb.2018.00751

**Published:** 2018-04-19

**Authors:** Chenggang Liu, Lei Jiang, Liangliang Liu, Li Sun, Wenjun Zhao, Yuqiu Chen, Tianming Qi, Zongxi Han, Yuhao Shao, Shengwang Liu, Deying Ma

**Affiliations:** ^1^College of Animal Science and Technology, Northeast Agricultural University, Harbin, China; ^2^Division of Avian Infectious Diseases, State Key Laboratory of Veterinary Biotechnology, Harbin Veterinary Research Institute, Chinese Academy of Agricultural Sciences, Harbin, China

**Keywords:** avian β-defensin 2, Newcastle disease virus, antiviral activity, ligands of toll-like receptors, p38 MAPK signaling pathway

## Abstract

The study was conducted to evaluate whether avian β-defensins (AvBDs) could be induced by Newcastle disease virus (NDV) infection, and to investigate the potential signaling pathway of AvBD2 induction in response to NDV infection as well. First, mRNA expression of AvBDs (1–14) was evaluated in the chicken embryo fibroblasts (CEFs) infected with NDV strain F48E9 at 6, 12, 24, 36, and 48 h post-inoculation (hpi), respectively. The results demonstrated a significant induction of AvBD2 in CEFs elicited by the NDV strain. Then, we expressed and purified the AvBD2 proteins in both eukaryotic cells and prokaryotic cells. Of the two recombinant AvBD2 proteins, only the protein expressed in eukaryotic cells showed directly antiviral activity against NDV strain F48E9 *in vitro*. Ligands of toll-like receptors (TLRs) were chosen as alternatives to NDV to further study signaling pathway of AvBD2 induction here, due to insufficient upregulation of AvBD2 expression elicited by NDV. We found that the mRNA expression of AvBD2 was highly upregulated by Pam3CSK4, FLA-ST, and ODN-M362. Then, four inhibitors of signaling pathway, including inhibitors of JNK, ERK1/2, p38 MAPK, and NF-κB, were used in this study. Of the four inhibitors, only inhibition of the p38 MAPK signaling pathway significantly reduced AvBD2 expression after stimulation with Pam3CSK4, FLA-ST and ODN-M362, respectively. Taken together, these results revealed that AvBD2 play a pivotal role in host innate immunity response to NDV infection. The mRNA expression of AvBD2 might be regulated in a p38 MAPK-dependent manner.

## Introduction

Newcastle disease virus (NDV), also known as avian paramyxovirus type 1, belongs to the genus *Avulavirus* in the family *paramyxoviridae* (Mayo, 2002) and is the etiological agent of Newcastle disease (ND), which is an important disease hazardous to the poultry industry. As a negative-sense, single-stranded, non-segmented, enveloped RNA virus, the NDV genome consists of 15,586 nucleotides (Liu et al., 2012). ND has a global distribution with a wide host range that not only results in high morbidity and mortality in poultry, but also has a great negative effect on the productivity of surviving birds (Wang et al., 2012). Numerous studies have focused on characterizing the pathogenesis of different NDV isolates in past years, while few studies have been done to evaluate host response to NDV infection. Our recent study demonstrated that NDV infection induces strong innate immune responses and intense inflammatory responses at early stage in goose (Xu et al., 2016). Similarly, it has been also reported that pigeon paramyxovirus type 1, a variant of NDV, induces immune responses characterized by activation of TLRs (TLR3 and TLR7), iNOS, and avian β-defensin (AvBD) 2 and 10 of pigeons post infection (Li et al., 2015).

The innate immune system is the first line of host defense against invading pathogens. Upon viral infection, the host cell detects the presence of viral components by means of germline-encoded pattern-recognition receptors (PRRs). In vertebrate species, three types of PRRs engage to recognize viruses: nucleotide oligomerization domain (NOD)-like receptors, Toll-like receptors (TLRs), and retinoic acid-inducible gene-1-like receptors (Yoneyama et al., 2004; Le Goffic et al., 2006; Allen et al., 2009; Barjesteh et al., 2016). TLRs are important PRRs that recognize essential components of microorganisms, such as membrane proteins, lipids, and nucleic acids (Rock et al., 1998). To date, at least 13 TLRs have been identified in mammals. Although TLR9 is absent and TLR8 is disrupted by a retroviral-like insertion element in avian species, other TLR orthologs have been described in avian species (Cheng et al., 2015). Additionally, two uncommon avian TLRs, TLR15 and TLR21, have been also identified (Keestra et al., 2010; Ramasamy et al., 2012). Interactions of TLRs with their specific ligands lead to an innate immune response through activation of MyD88-dependent or -independent intracellular pathways to activate the transcriptional factors mitogen-activated protein kinase (MAPK) and nuclear factor-*kappa* B (NF-κB), resulting in the expression of host defense peptides (HDPs, also known as antimicrobial peptides) and cytokines (Sato et al., 2005; Yamamoto and Takeda, 2010; Abdel-Mageed et al., 2014).

The MAPK signal transduction pathways are evolutionarily conserved in eukaryotes and involved in many cellular processes, including immune response, apoptosis, and proliferation (Chang and Karin, 2001; Whitmarsh, 2007; Kyriakis and Avruch, 2012). The MAPK family of serine/threonine kinases consists of at least three subfamilies: c-Jun N-terminal kinase (JNK), p38 MAPK, and extracellular signal-regulated kinase 1/2 (ERK1/2) (Kogut et al., 2012). As a regulator of the transcription factor c-Jun and a mediator of intra- or extra-cellular stress, the JNK cascade is the stress-activated protein kinase cascade (Davis, 1994; Plotnikov et al., 2011). The p38 cascade is another MAPK pathway that demonstrates considerable cross-talk and shares components with the other stress-induced cascade of JNKs (Plotnikov et al., 2011). The ERK1/2 cascade was the first MAPK pathway elucidated (Seger and Krebs, 1995). In mammalian cells, HDPs could be induced by activation of MAPKs during stimulation by extracellular stress signals (Krisanaprakornkit et al., 2002; Lee et al., 2008; Lewis et al., 2016).

The HDPs are important components of the innate immune response. Being the most studied family of cysteine-rich HDPs, defensins are critical to innate immunity and subsequent protection against infection (Yang et al., 1999). They can be divided into three subfamilies, named α, β, and θ-defensins according to their structural properties (Ganz, 2003). α-Defensins are only present in mammalian species and form disulfide bridges between Cys^1^–Cys^6^, Cys^2^–Cys^4^, and Cys^3^–Cys^5^. β-Defensins can be found in all vertebrate species and form disulfide bridges between Cys^1^–Cys^5^, Cys^2^–Cys^4^, and Cys^3^–Cys^6^. θ-Defensins are cyclic defensins, with cystine bridges between Cys^1^-Cys^6^, Cys^2^-Cys^5^, and Cys^3^-Cys^4^ and are found in rhesus monkeys and baboons (Lehrer and Ganz, 2002; Yang et al., 2004; Klotman and Chang, 2006; Lehrer et al., 2012; Cuperus et al., 2013). Of the three defensin subfamilies, only β-defensins have been found in birds (Cuperus et al., 2013). In addition to antibacterial activities, defensins from various species have been demonstrated previously to display antiviral activity. It has been reported that human β-defensins show direct inhibitory activities against various viruses, including human immunodeficiency virus (HIV) (Quinones-Mateu et al., 2003), adeno-associated virus (Virella-Lowell et al., 2000), adenovirus (Bastian and Schafer, 2001), influenza virus (Leikina et al., 2005), and respiratory viruses (Zhao et al., 2016). It is also reported that rainbow trout β-defensin exhibit antiviral activity against the enveloped viral hemorrhagic septicemia virus (Falco et al., 2008). In addition to their direct antimicrobial activity, some β-defensins are capable of promoting local innate and systemic adaptive immune responses (Yang et al., 1999; Barabas et al., 2013; Cuperus et al., 2013). It has been shown that β-defensins from various species can be induced by various viruses following infection both *in vivo* and *in vitro* (Grubor et al., 2004; Chong et al., 2008; Ma et al., 2011). Sheep β-defensin-1 was increased following parainfluenza virus type 3 infection (Grubor et al., 2004). In mice, influenza virus could induce β-defensin-1, -2, and -3 upregulation in the lungs (Chong et al., 2006). Human epithelial cells highly expressed β-defensins when infected virus (Proud et al., 2004). To date, 14 AvBDs have been identified in chicken (Lynn et al., 2007). In addition to their antibacterial potential, AvBDs have also been demonstrated direct antiviral activity against various viruses, including pigeon *Paramyxovirus* type 1 (Li et al., 2015), infectious bronchitis virus (IBV) (Xu et al., 2015), and duck hepatitis virus (DHV) (Ma et al., 2012).

Based on the antiviral activity of AvBDs and its importance in the response to viral infections, as well as the need for new antiviral drugs, the purpose of this study was to assess induction of AvBD2 by NDV infection, and then speculate the signaling pathway of AvBD production.

## Materials and Methods

### Ethics Statement

All animal experimental procedures were approved by the Ethical and Animal Welfare Committee of Heilongjiang Province, China (License No. SQ20160408).

### Eggs, Cells Culture, and Virus Strains

Specific-pathogen-free (SPF) embryonated chicken eggs were supplied by the Harbin Veterinary Research Institute, Chinese Academy of Agricultural Sciences (Harbin, China). Chicken embryo fibroblasts (CEFs) were prepared with 9-day-old SPF embryonated chicken eggs as previously described (Shahsavandi et al., 2013). The CEFs were cultivated in Dulbecco’s modified Eagle’s medium (DMEM) supplemented with 10% fetal bovine serum (FBS), 100 U/ml penicillin, 100 μg/ml streptomycin, and 2 mM L-glutamine, at 37°C in a humidified atmosphere of 95% air/5% CO_2_. Expi293 FreeStyle cells were incubated at 37°C in a humidified atmosphere of 90% air/10% CO_2_ on an orbital shaker platform rotating at 125 rpm and then maintained in Expi293 Expression medium (Gibco, Carlsbad, CA, United States). The virulent NDV strain F48E9 was used in this study in the biosafety level-3 laboratory (Guo et al., 2014). The intracerebral pathogenicity index of the NDV strain is 2.0 (Guo et al., 2014). The median cell culture infective dose (TCID_50_) of the virus in CEFs was 10^-6.75^/100 μl (tested in this study).

### Infection of CEFs With NDV Strain F48E9

The CEFs were cultured at an initial density of 1 × 10^4^ cells per well in a 96-well cell culture plate and kept overnight. Firstly, survival rate of CEFs infected with NDV was measured by the Cell Counting Kit-8 (CCK-8) assay according to the manufacturer’s protocol. In brief, CEFs in different multiplicity of infection (MOI) (0.01, 0.1, 1, 10, and 100) at different time points (24, 36, and 48 h post-infection, hpi) was added to the CCK-8 solution (5 mg/ml, 10 μl/well) before incubation at 37°C for 1 h. Absorbance was then measured at a wavelength of 450 nm by using a microplate reader (model 680; Bio-Rad Laboratories, Hercules, CA, United States). Then, CEFs were cultured in 12-well cell culture plates at a density of 1 × 10^6^ cells per well, and were used at 90% confluence. The cells were infected with NDV strain F48E9 at MOI of 1 at 37°C for 1.5 h. After an absorption period of 1.5 h, the wells were supplemented with DMEM containing 2% FBS after washing off the unattached virus. The cytopathic effect (CPE) was observed for 48 h in succession (Li et al., 2014). At 6, 12, 24, 36, and 48 hpi, the supernatants of the cell cultures were harvested and assayed for virus titer using the hemagglutination (HA) and TCID_50_, as previously described. Both the infected and uninfected cells were harvested for RNA isolation. Uninfected cells were used as negative controls in these experiments. The experiment was performed in triplicate. The challenge test was conducted in isolators in biosafety level-3 facilities under negative pressure.

### HA and TCID_50_ Assay

The HA assay was performed according to standard method as described by reference (Sano and Ogawa, 2014). The end-point dilution is known as 1 HA unit (HAU) and the number of HAUs in each 50 μl is the reciprocal of the highest dilution (Sano and Ogawa, 2014). The TCID_50_ of NDV F48E9 strain in CEFs was calculated by using the Reed and Muench method (Reed and Muench, 1938). The experiment was performed in triplicate.

### RNA Extraction and RT-PCR Analysis

The RNA extraction and One-step Real-Time (RT)-PCR were conducted as described in previous studies (Xu et al., 2015, 2016). Primers of 18S rRNA and AvBDs (1–14) used in this study were described by reference (Xu et al., 2015).

### Preparation of AvBD2 Protein in Eukaryotic Cells

The pCAG (+)-AvBD2-His plasmid, in which AvBD2 cDNA was in frame fused to the C-terminal His tag in the pCAG (+) vector plasmid (stored in our laboratory), was constructed by standard molecular biology techniques. Briefly, cDNA sequences that encoded AvBD2 bases (GenBank Accession No. NM_204992.2) (amino acid sequence: RDMLFCKGGSCHFGGCPSHLIKVGSCFGFRSCCKWPWNA) were amplified by PCR from the plasmid of pProEX-HTa-AvBD2 (Xu et al., 2015) using the following primers: forward, 5′–CCG*CT* *CGAG*GCCACCATGAGGATTCTTTACCTGCTTT–3′; reverse, 5′–CG*GGATCC*TTAATGGTGATGGTGATGATGTGCATTCCAAGGCCATTTG–3′. The PCR products flanked by the *Xho* I and *Bam* HI restriction sites were inserted into the same sites of the pCAG (+) vector. The resulting constructs were confirmed to contain AvBD2 by sequencing and were then transformed into Expi293 cells using the ExpiFectamine 293 Transfection system. A total of 5 days after transfection, the cells were harvested by centrifugation (750 × *g*, 25°C, 5 min) and then lysed by RIPA Lysis Buffer (Beyotime, Shanghai, China). After centrifugation (13,000 × *g*, 4°C, 30 min), the supernatant, including the soluble AvBD2 protein, was purified using the Ni-NTA Purification System (Invitrogen), and the purified AvBD2 protein was desalted using the Amicon^®^ Ultra-15 3K Centrifugal Filter Device (EMD Millipore, Temecula, CA, United States). The released mature AvBD2 was examined by 12% SDS–PAGE (Li et al., 2015). The concentration of the protein was measured using the NanoVue Plus spectrophotometer (GE Healthcare, Cleveland, OH, United States). Then, AvBD2 protein band was cut out and analyzed by mass spectrometry (Beijing Protein Innovation Co., Ltd., Beijing, China).

### Preparation of AvBD2 Protein in Prokaryotic Cells

The construct of pProEX-HTa-AvBD2 plasmid (Xu et al., 2015) was transformed into *Escherichia coli* BL21 (DE3) competent cells and induced as described previously (Xu et al., 2015). The protein was expressed, purified and refolded as described previously (Xu et al., 2015). Purified AvBD2 protein was desalted using the 3K centrifugal filter device. The protein was examined by 12% SDS-PAGE (Li et al., 2015). The concentration of the protein was measured using the NanoVue Plus spectrophotometer (GE Healthcare, Cleveland, OH, USA).

### Antiviral Activity of Recombinant AvBD2 Proteins Against NDV *in Vitro*

Three experiments were conducted to evaluate antiviral activities of both recombinant AvBD2 proteins against NDV by using CCK-8 assay (Gong et al., 2010). (1) The NDV strain (1 MOI) was pre-incubated with various concentrations of AvBD2 (3.75-120 ng/μl) freshly diluted in PB for1.5 h at 37°C. To begin infection, the cells were washed twice with PBS in order to remove FBS, followed by adding 100 μl of the AvBD2 protein-virus mixture and further incubated for1.5 h at 37°C. (2) CEFs were infected with NDV at MOI of 1 and incubated for 1.5 h at 37°C, followed by addition of various concentrations of AvBD2 (3.75-120 ng/μl) further incubated for 1.5 h at 37°C. (3) AvBD2 protein (3.75-120 ng/μl) was incubated with CEFs for 1.5 h at 37°C, followed by addition of NDV (1 MOI) and incubation for another 1.5 h at 37°C. Then, the cells were washed two times with PBS and 100 μl of fresh DMEM was added to each well and plates were incubated at 37°C for 48 h. Ten μl of CCK-8 solution (5 mg/ml) was added into each well of the cell culture plate and incubated further for 40 min at 37 °C. The absorbance at 450 nm was measured as described above. Cells without virus and AvBD2 mixture were used as a negative control. Cells infected with the virus only served as a positive control. The antiviral activity of AvBD2 was calculated as follows: protective rate = [(mean optical density of test – mean optical density of positive controls) / (optical density of negative controls – mean optical density of positive controls)] × 100% (Pauwels et al., 1988; Gong et al., 2010). The experiments were performed in triplicate. Furthermore, the IC_50_ value was calculated based on the log values by using GraphPad Prism 5 software in the present study (Motulsky, 2007).

### Stimulation of CEFs With TLR Ligands

The CEFs were cultured in 12-well cell culture plates and were used at 90% confluence. All TLR ligands were purchased from InvivoGen (San Diego, CA, USA) and were dissolved in double distilled H_2_O. The cells were stimulated with Pam3CSK4 (synthetic triacylated lipoprotein) (0.4 μg/ml), LPS (standard preparation of lipopolysaccharide from *E. coli* 055: B5) (2 μg/ml), poly I:C (polyinosinic-polycytidylic acid) (50 μg/ml), FLA-ST (flagellin from *Salmonella typhimurium*) (2 μg/ml), R848 (imidazoquinoline compound) (5 μg/ml), and ODN-M362 (class C CpG oligonucleotide) (2.5 μM), respectively, and then harvested at 6, 24, and 48 h post-stimulation (hpt) for RNA isolation and AvBD2 mRNA evaluation as described above. The negative controls were treated with the same volume of endotoxin-free H_2_O. All experiments were performed in triplicate.

### Inhibition Assay of Signaling Pathway

The CEFs were cultured in 12-well cell culture plates and were used at 90% confluence. CEFs were pre-treated for 1 h with various inhibitors (Beyotime, Shanghai, China) of the signaling pathway dissolved in dimethyl sulfoxide (DMSO). The following concentration were used: JNK inhibitor (SP600125), 50 μM; ERK1/2 inhibitor (PD98059), 50 μM; p38 MAPK inhibitor (SB203580), 50 μM; NF-κB inhibitor (PDTC), 50 μM; the negative control (DMSO, volume < 0.1%), and an untreated control, respectively. Two experiments were conducted to evaluate inhibition of the signaling pathway on AvBD2 expression elicited by either of NDV, or ligands of TLRs. After pre-treatment with inhibitors for 1 h, (1) The cells were infected with NDV (1 MOI) at 37°C for 1.5 h. The wells were supplemented with DMEM containing 2% FBS after washing off the unattached virus and the CEFs were cultured for another 36 or 48 h. (2) Either of ODN-M362 (2.5 μM), FLA-ST (2 μg/ml), or Pam3CSK4 (0.4 μg/ml) were added to the plates and the CEFs were cultured for another 24 h. Then, the cells were harvested for RNA isolation and AvBD2 mRNA evaluation as described above. All experiments were performed in triplicate.

### Statistical Analysis

Statistical analysis was used GraphPad Prism software v5.01 (GraphPad Software Inc., San Diego, CA, United States). One-way analysis of variance followed by the Tukey–Kramer post-test was used. A *p* < 0.05 was considered statistically significant.

## Results

### Biological Characterization of NDV Strain F48E9 Infection in CEFs

**FIGURE 1:**
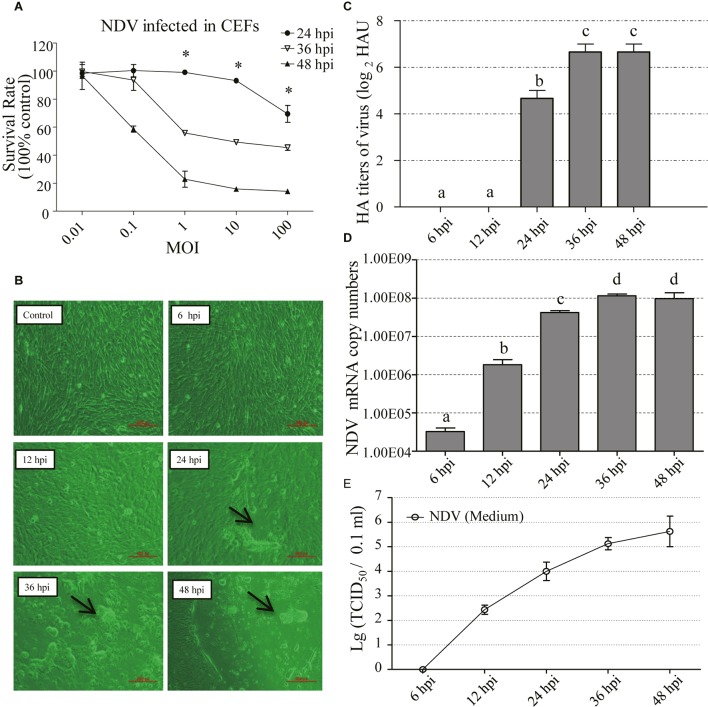
Biological characterization of NDV-F48E9 infection in CEFs. **(A)** Survival rate of NDV infected CEFs in different MOI at different time points. CCK-8 was used to detect cell viability. Absorbance was measured at a wavelength of 450 nm by using a microplate reader (model 680; Bio-Rad Laboratories, Hercules, CA, United States). Protective rate was described as previous reports (Pauwels et al., 1988; Gong et al., 2010). Each group has three replicates. The difference between the same MOI at three different infection time points is represented by ^∗^*p* < 0.05. **(B)** Cytopathic effect of NDV at 6, 12, 24, 36, and 48 hpi. Black arrow indicated the syncytia induced by NDV. **(C)** HA titer of the supernatants of NDV-infected cells. **(D)** Viral RNA copy numbers measured by RT-PCR in CEFs infected with NDV. **(E)** TCID_50_ of the supernatants of NDV-infected cells. All assays were performed in triplicates. Each bar is the mean ± SEM. ^a,b,c,d^The values with different letters are significantly different (*p* < 0.05)

The CEFs were infected with NDV strain F48E9 at different MOI (0.01, 0.1, 1, 10, and 100). Cell survival rate was measured using the CCK-8 assay at different hours post-infection (24, 36, and 48 hpi). The results showed that the survival rate of cells was significantly increased in treatments of 1–100 MOI of NDV infection at 24 hpi, compared to the other treatments (*p* < 0.05) (**Figure 1A**). Then, the CPE (1 MOI) was monitored for 48 h. The results showed no obvious CPE in NDV-infected CEFs until 24 hpi. The CPE was characterized by syncytia owing to coalescence with adjacent infected cells (**Figure 1B**). Similarly, HA titers of cell supernatants were undetectable at 6 and 12 hpi, but it was detectable at 24 hpi and peaked at 48 hpi (**Figure 1C**). To confirm the presence of NDV in the infected cells, viral loads of NDV in CEFs were examined by RT-PCR (**Figure 1D**). Viral replication was time-dependent, occurring as early as 6 hpi, and peaked at 36 hpi. In contrast, no viral RNA was detected in the uninfected cells (data not shown). Collectively, these results confirmed successful NDV infection of cells. Furthermore, the supernatant was tested for TCID_50_ in CEF and the result showed that the titers were detectable at 12 hpi and reached the highest at 48 hpi (**Figure 1E**).

### Changes in mRNA Expression of AvBDs in CEFs in Response to Infection With NDV Strain F48E9

**FIGURE 2:**
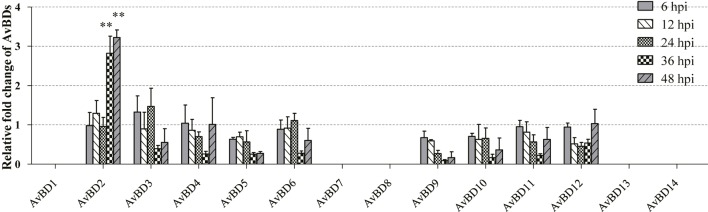
Relative gene expression of AvBDs in response to NDV infection in CEFs. Fold change of mRNA expression in CEFs of each group were measured by RT-PCR at 6, 12, 24, 36, and 48 hpi, respectively. Transcript levels of AvBDs were normalized to 18S rRNA and mRNA expression is shown as the fold change relative to the control. All assays were performed in triplicate, and each bar is the mean ± SEM. Statistically significant differences between groups are indicated by ^∗^*p* < 0.05 or ^∗∗^*p* < 0.01.

In order to determine whether mRNA expression of AvBDs in CEFs was induced in response to NDV infection, expression of AvBDs 1–14 in the CEFs was evaluated at 6, 12, 24, 36, and 48 hpi, respectively, using RT-PCR. In general, most of the AvBDs measured were detectable in both the control and the infected CEFs at each time point. In contrast, little expression of AvBD1, 7, 8, 13, and 14 was detected in CEFs during the experimental period (**Figure 2**). Interestingly, of the 14 AvBDs, only AvBD2 expression was significantly upregulated in CEFs at 36 hpi, and peaked at 48 hpi, as compared with the control (*p* < 0.05). However, upregulation of the other AvBDs expression was less significant in NDV infected CEFs at each time point, as compared to the control (*p* > 0.05).

### Antiviral Activity of Recombinant AvBD2 Protein Against NDV *in Vitro*

**FIGURE 3:**
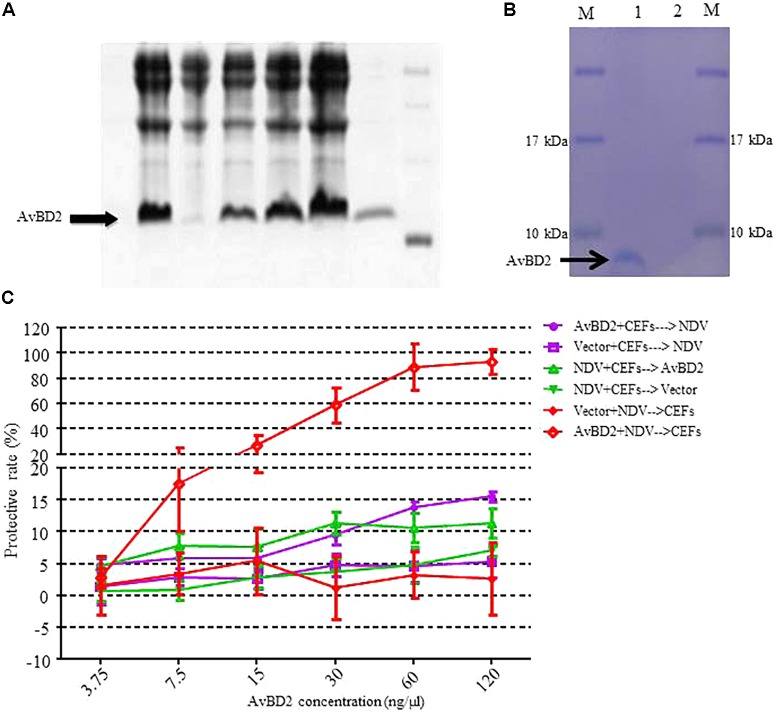
Recombinant expression of AvBD2 protein and antiviral activity of AvBD2 against NDV. **(A)** 12% SDS–PAGE analysis of His-tagged recombinant AvBD2 protein expressed in *E*. *coli* BL21 (DE3) cells. *Lane* 1: Supernatant; *lane* 2: Inclusion body; *lane* 3: Total protein of Rosetta containing AvBD2 without IPTG induction; *lanes* 4–6: Total protein of Rosetta containing AvBD2 at 2, 4, 6 h after induction with IPTG, respectively; *lane* 7: Purified recombinant AvBD2 protein; *lane* M: Protein marker. **(B)** 12% SDS–PAGE analysis of His-tagged AvBD2 protein expressed in Expi293 cells. *Lane* 1: purified AvBD2 with a His-tag on the C-terminal; *lane* 2: purified vector with a His-tag on the C-terminal; *lane* M: protein molecular weight marker. **(C)** Protection of CEFs from NDV infection by using the CCK-8 assay. *Red curve*, NDV (1 MOI) and AvBD2 protein (3.75–120 ng/μl) were mixed for 1.5 h at 37°C. Cells were washed and then incubated with the NDV-AvBD2 protein mixture for another 1.5 h at 37°C. *Green curve*, NDV (1 MOI) and equal volume of PB were mixed and then incubated with CEFs for 1.5 h at 37°C. Cells were washed and incubated with AvBD2 protein and equal volume of DMEM without serum for another 1.5 h. *Purple curve*, AvBD2 protein (3.75–120 ng/μl) and equal volume of DMEM without serum were mixed to incubate with CEFs for 1.5 h at 37°C. Cells were washed and then the mixtures of NDV (1 MOI) and equal volume of PB were added and incubated with CEFs for another 1.5 h at 37°C. Then, the cells were washed, and 2% DMEM were added and incubated for 48 h at 37°C. Then CCK-8 solution (5 mg/ml, 10 μl/well) was added to the plate. The absorbance at 450 nm was measured using a microplate reader after the incubation at 37°C for 1.5 h. The vector was used as control for each group. Cells without virus-protein mixture were also used as a negative control. Cells only infected with the virus served as a positive control. The experiment was performed in triplicate.

In order to evaluate the antiviral activity of AvBD2 against NDV *in vitro*, recombinant AvBD2 proteins were expressed and purified in both prokaryotic cells (**Figure 3A**) and eukaryotic cells (**Figure 3B**). The two kinds of recombinant AvBD2 protein sample were separated on 12% SDS–PAGE. The gel band was then confirmed by mass spectrometry. The result showed that the molecular weight of the recombinant protein was approximately 7.5 kDa, and pI value was 9.38. The Mascot Score of the gel band confirmed by mass spectrometry was 342. Proteins with significant score of > 21 (*p* < 0.05) were considered successfully identified. This result confirmed that AvBD2 protein was successfully expressed in eukaryotic cells. Detailed information about the result of mass spectrometry is shown in **Table 1**.

**Table 1:** Analyses of AvBD2 by mass spectrometry.

Accession No.^a^	Ion scores^b^	Matched peptides^c^	Sequence coverage	Mass	pI value	Protein name
P46158	342	RDMLFCK VGSCFGFR	23%	7479	9.38	Gallinacin-2 OS = Gallus gallus GN = GAL2 PE = 1 SV = 2

^a^*Accession No. is according to Uniprot database. ^*b*^Ion scores were from LC-MS/MS identification. Proteins with significant ion score of > 21 (*p* < 0.05) were considered successfully identified. ^*c*^Peptides identified by LC-MS/MS matched the successfully identified protein*.

To evaluate the antiviral activity of the two kinds of recombinant AvBD2 proteins against NDV, three experiments were conducted by measuring cell viability with the CCK-8 assay. The protective rate of cells is significantly increased in the treatment in which NDV was pre-incubated with AvBD2 expressed in eukaryotic cells, compared to that of the other two treatments. The result demonstrated that the recombinant AvBD2 showed directly antiviral activity against the NDV strain. The protective rate reached 93.35% at the concentration of 120 ng/μl (**Figure 3C**). The IC_50_ of the recombinant AvBD2 was 19.61 ± 4.76. In addition, the results of the cytotoxicity by CCK-8 assay indicated that the recombinant AvBD2 showed little cytotoxicity in CEFs, even at concentration of 120 ng/μl (data not shown). In contrast, little significant protection against NDV was found by recombinant AvBD2 expressed in prokaryotic cells (even at concentration of 300 ng/μl), compared with the control group (data not shown).

### Effect of MAPKs/NF-κB Signaling Pathway Inhibitors on AvBD2 Expression in CEFs Infected With NDV

**FIGURE 4:**
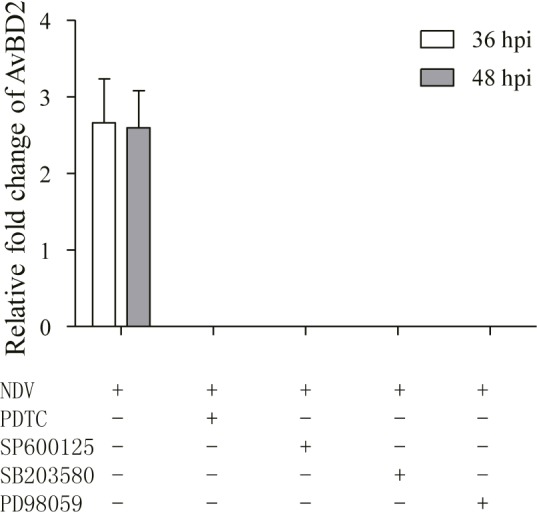
Inhibitors of MAPK/NF-κB inhibit AvBD2 expression mediated by NDV. CEFs were pre-treated with 50 μM PDTC (NF-κB inhibitor), 50 μM SP600125 (JNK inhibitor), 50 μM SB203580 (p38 MAPK inhibitor), or 50 μM PD98059 (ERK1/2 inhibitor) for 1 h, then infected with or without NDV (1 MOI) for another 36 or 48 h. Fold change of mRNA expression in CEFs of each group were measured at 36 or 48 hpi using RT-PCR. Transcript level of AvBD2 was normalized to the levels of 18S rRNA in the same samples and gene expression is presented as the fold change relative to the uninfected group. All assays were performed in triplicate, and each bar is the mean ± SEM.

In order to elucidate the regulatory mechanism of AvBD2 expression in response to NDV infection, CEFs were infected with NDV for 36 and 48 h after pre-treatment with MAPK and NF-κB pathway inhibitors, respectively. However, AvBD2 mRNA expression was not detectable at both time points (**Figure 4**), which may be due to insufficient upregulation of AvBD2 mRNA expression induced by NDV (the fold change relative to the uninfected group is less to 3). Alternatives to NDV, such as ligands of TLRs, needed to be identified for further study.

### Regulation of AvBD2 Expression in CEFs by TLR Ligands

**FIGURE 5:**
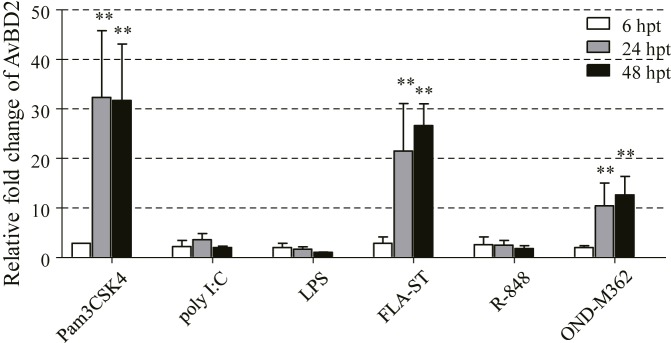
Induction of AvBD2 mRNA expression following TLR ligands stimulation in CEFs. CEFs were treated with Pam3CSK4 (0.4 μg/ml), polyI:C (50 μg/ml), LPS (2 μg/ml), FLA-ST (2 μg/ml), R848 (5 μg/ml), and ODN-M362 (2.5 μM), respectively. CEFs in the control groups received medium. Fold change of mRNA expression in CEFs of each group were measured at 6, 24, and 48 h post-treatment using RT-PCR. Transcript levels of AvBD2 were normalized to 18s rRNA and mRNA expression is presented as the fold change relative to the control group. All assays were performed in triplicate, and each bar is the mean ± SEM. Statistical significant difference between cells without and with TLR ligands are indicated by ^∗^ *p* < 0.05 or ^∗∗^*p* < 0.01.

In order to further understand the molecular basis of the regulation of AvBD2 secretion in response to NDV infection, ligands of TLRs were chosen as alternatives to NDV in the study. Different TLR ligands, namely, Pam3CSK4 (TLR1/2), polyI:C (TLR3), LPS (TLR4), FLA-ST (TLR5), R848 (TLR7), and ODN-M362 (TLR21) were employed in this study. The mRNA expression of AvBD2 was evaluated in CEFs stimulated with each TLR ligands at 6, 24, and 48 hpt, respectively. The results demonstrated that mRNA expression of AvBD2 in CEFs was significantly up-regulated by Pam3CSK4, FLA-ST, and ODN-M362 stimulation at both 24 and 48 hpi, compared with respective control (**Figure 5**). In contrast, little significant regulation on AvBD2 expression was observed by polyI:C, LPS, and R848 in this study.

### The p38 MAPK Signaling Pathway Plays an Essential Role in AvBD2 Expression Induced by TLR Ligands

**FIGURE 6:**
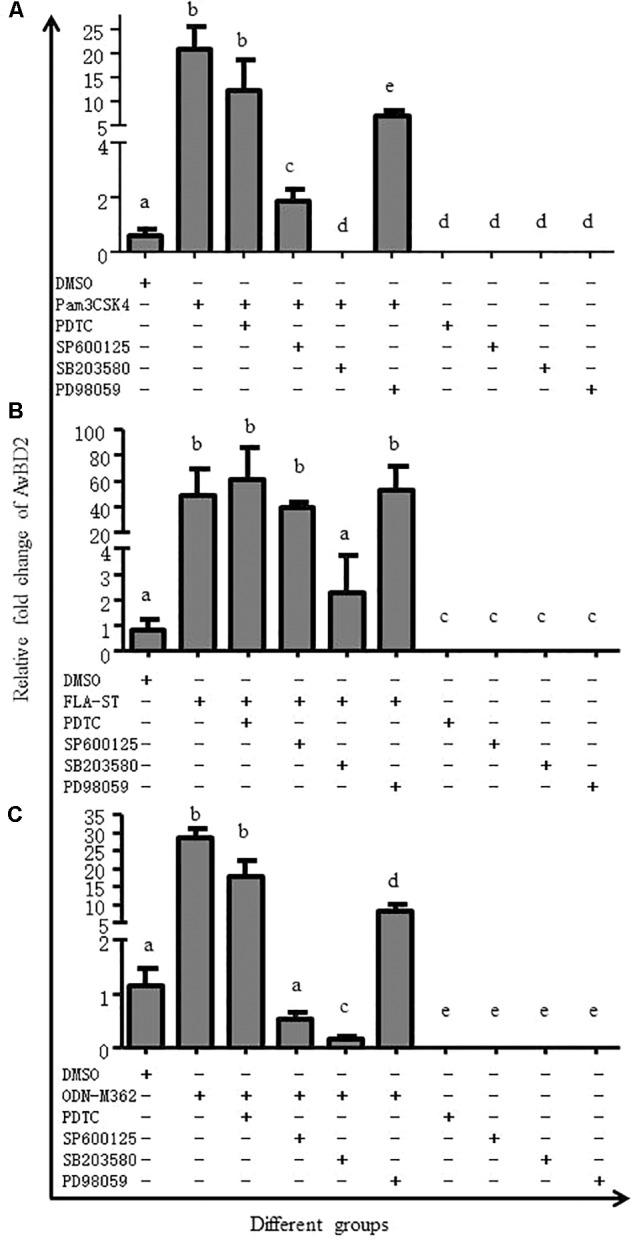
MAPK signaling pathway is involved in AvBD2 induction mediated by TLR ligands. CEFs were pre-treated with 50 μM PDTC (NF-κB inhibitor), 50 μM SP600125 (JNK inhibitor), 50 μM SB203580 (p38 MAPK inhibitor), or 50 μM PD98059 (ERK1/2 inhibitor) for 1 h, followed by stimulation with or without **(A)** Pam3CSK4 (0.4 μg/ml), **(B)** FLA-ST (2 μg/ml), or **(C)** ODN-M362 (2.5 μM) for another 24 h. The DMSO group was used as a negative control. Fold change of mRNA expression in CEFs of each group were measured by RT-PCR at 24 h post-treatment. Transcript level of AvBD2 was normalized to the levels of 18S rRNA in the same samples and gene expression is presented as the fold change relative to the control group. All assays were performed in triplicate, and each bar is the mean ± SEM. ^a,b,c,d,e^The values with different letters are significantly different (*p* < 0.05).

In order to elucidate which signaling pathway regulates secretion of AvBD2, we have succeeded in detecting ERK, p38, JNK kinases, and NF-κB 1 in CEFs in this study. Then, inhibition of transcriptional factor in CEFs was performed. As shown in **Figure 6**, expression of AvBD2 was significantly inhibited by SB203580 in TLR ligands-induced cells (*p* < 0.05) (including Pam3CSK4, FLA-ST, and ODN-M362, respectively) (**Figures 6A–C**). Both SP600125 and PD98059 had only a partial effect on AvBD2 expression (**Figures 6A,C**). However, the mRNA expression of AvBD2 was undetectable in which only be pre-treated with various inhibitors (no TLR ligands added) (**Figures 6A–C**). These results suggested that the p38 MAPK signaling pathway exhibited a more important role on AvBD2 expression than both the ERK1/2 and JNK signaling pathways (*p* < 0.05). In addition, PDTC treatment had little effect on AvBD2 expression (**Figures 6A–C**). Together, these data revealed that transcription factor p38 MAPK may play an important role in the regulation of AvBD2 production in response to stimulation with the TLR ligands (Pam3CSK4, FLA-ST, and ODN-M362).

## Discussion

The ND is an infectious, highly contagious and pathogenic avian viral disease caused by virulent NDV strains. Despite considerable understanding of the pathogenesis of the virus, a little is known about the host-virus interaction. Recent studies have shown upregulation of AvBDs, cytokines and iNOS in goose and chickens infected with virulent NDV, respectively (Ecco et al., 2011; Rasoli et al., 2014; Li et al., 2015; Xu et al., 2016). However, limited researches have been reported on mechanism of AvBD induction in chickens infected with NDV. It is well documented that HDPs, including AvBDs, are key components of the host innate immune response against pathogens and potentially provide a link between innate and adaptive immunity (Cuperus et al., 2013; Garcia-Valtanen et al., 2014). The present study showed that only AvBD2 expression was significantly upregulated in CEFs in response to NDV infection *in vitro*, comparing to those of other 13 AvBDs. Consistent with this observation, our previous reports also showed that AvBDs, including AvBD2, from various avian species can be induced by several viruses, including DHV (Ma et al., 2011, 2012), IBV (Xu et al., 2015), PPMV-1 (Li et al., 2015), and a genotype VIId NDV (Xu et al., 2016) strain of goose origin (go/CH/LHLJ/1/06) *in vivo*. Overall, these studies suggested that AvBDs, especially AvBD2, are key components of the innate response and may play pivotal role in the host defense against viral infection.

Furthermore, the current results also demonstrated that the recombinant AvBD2 expressed in eukaryotic cells had directly antiviral activity against the NDV strain *in vitro*. Consistent with the present results, the antiviral activity of other HDPs against different viruses, including human α-defensins (HNP1–4, HD5, and HD6) against Herpes Simplex Virus (HSV), Rabbit α-defensins against HSV-1 and -2 (Lehrer et al., 1985), and Rhesus θ-defensin 2 against HSV-2 (Yasin et al., 2004), has been well documented in past years. The antiviral activity of β-defensins from different species against viruses, including HSV (Hazrati et al., 2006), spring viremia of carp virus (Garcia-Valtanen et al., 2014), HIV-1 (Quinones-Mateu et al., 2003), parainfluenza virus 3 (Grubor et al., 2004), and influenza virus (Gong et al., 2010) have also been described previously. Similarly, several AvBDs, including AvBD2, exhibited directly antiviral activity against DHV (Ma et al., 2011, 2012), IBV (Xu et al., 2015), and PPMV-1 (Li et al., 2015) in our recent studies. These findings further confirmed the importance of HDPs in the host defense response against viral infection and open the possibility of developing novel agents based on HDPs to treat viral infections.

Notably, in contrast to the recombinant AvBD2 protein expressed in eukaryotic cells, the recombinant AvBD2 protein expressed in prokaryotic cells exhibited little directly antiviral activity against NDV in this study. The results were partly different from previous study on the other virus (Xu et al., 2015). The possible reason needed to be further study. It is considered that structural organization of most defensins is essential for their antimicrobial activity (Hoover et al., 2003). Previous study showed that antimicrobial activity of linear AvBD2-Acm protein is lower than the fully oxidized protein, indicating the critical role of the tertiary structure on antimicrobial activity (Derache et al., 2012). Eukaryotic cell expression system could provide post-translational modifications, such as glycosylation and correct protein folding, which is critical to biological activity of protein (de la Salle et al., 1985). In this study, Expi293 FreeStyle cells were used to transfect pCAG (+)-AvBD2-His plasmid in the eukaryotic expression system. The antiviral activity of the recombinant AvBD2 against NDV might be related to its fully modified and folded tertiary structure.

The MAPK family is responsible for important signaling pathways which regulate immune responses (Xing et al., 2010). It was reported that the responses of HDPs to TLR ligands rely on the NF-κB or/and MAPK signaling pathways in mammals *in vitro* (Frigo et al., 2004; Roux and Blenis, 2004, Lee et al., 2008; Han et al., 2009, Lewis et al., 2016). Previous study also reported that avian influenza virus activates ERK, p38 and JNK in avian species (Xing et al., 2010). In this study, we found that AvBD2 expression was significantly suppressed by inhibitor of p38 MAPK. The results revealed that AvBD2 expression might mainly responds to TLR ligands through the p38 MAPK signaling pathway. Consistent with the current results, it was also reported that, in most cases, regulation of TLRs was independent of NF-κB but dependent on one or more of the MAPK pathway components (Krisanaprakornkit et al., 2002; Peroval et al., 2013). Similarly, inhibition of p38 MAPK by SB203580 could abolish HBD2 up-regulation by NTHi in human middle ear epithelial cells (Lee et al., 2008). These results showed that β-defensin 2 has a similar regulatory mechanism in different species.

## Conclusion

This study reports that the mRNA expression of AvBD2 was upregulated in CEFs by NDV strain F48E9 infection *in vitro* and the recombinant AvBD2 protein expressed by eukaryotic cells demonstrated direct antiviral activity against the NDV strain. The AvBD2 expression is also induced by ligands of TLRs (Pam3CSK4, FLA-ST, and ODN-M362), and suppressed by inhibitor of p38 MAPK. Our results revealed that AvBD2 play a pivotal role in host innate immunity response to NDV infection. The AvBD2 expression might be regulated in a p38 MAPK-dependent manner.

## Author Contributions

CL performed the experiments and wrote the manuscript. LJ, LL, WZ, YC, and TQ performed the experiments and the calculation. LS, ZH, and YS collected the samples. DM and SL designed and conducted the study, and wrote the manuscript.

## Conflict of Interest Statement

The authors declare that the research was conducted in the absence of any commercial or financial relationships that could be construed as a potential conflict of interest.

## References

[B1] Abdel-Mageed A. M. Isobe N. Yoshimura Y. (2014). Effects of different TLR ligands on the expression of proinflammatory cytokines and avian beta-defensins in the uterine and vaginal tissues of laying hens. *Vet. Immunol. Immunopathol.* 162 132–141. 10.1016/j.vetimm.2014.10.013 25467889

[B2] Allen I. C. Scull M. A. Moore C. B. Holl E. K. McElvania-TeKippe E. Taxman D. J. (2009). The NLRP3 inflammasome mediates in vivo innate immunity to influenza A virus through recognition of viral RNA. *Immunity* 30 556–565. 10.1016/j.immuni.2009.02.005 19362020 PMC2803103

[B3] Barabas N. Röhrl J. Holler E. Hehlgans T. (2013). Beta-defensins activate macrophages and synergize in pro-inflammatory cytokine expression induced by TLR ligands. *Immunobiology* 218 1005–1011. 10.1016/j.imbio.2012.11.007 23332217

[B4] Barjesteh N. Alkie T. N. Hodgins D. C. Nagy E. Sharif S. (2016). Local innate responses to TLR ligands in the chicken trachea. *Viruses* 8:207. 10.3390/v8070207 27455308 PMC4974541

[B5] Bastian A. Schafer H. (2001). Human alpha-defensin 1 (HNP-1) inhibits adenoviral infection in vitro. *Regul. Pept.* 101 157–161. 10.1016/S0167-0115(01)00282-8 11495691

[B6] Chang L. Karin M. (2001). Mammalian MAP kinase signalling cascades. *Nature* 410 37–40. 10.1038/35065000 11242034

[B7] Cheng Y. Wang H. Yan Y. Ding C. Sun J. (2015). Two myeloid differentiation factor 88 (MyD88) isoforms identified in ducks. *Dev. Comp. Immunol.* 52 144–154. 10.1016/j.dci.2015.03.015 26004012

[B8] Chong K. T. Thangavel R. R. Tang X. H. (2008). Enhanced expression of murine beta-defensins (MBD-1,-2,-3, and -4) in upper and lower airway mucosa of influenza virus infected mice. *Virology* 380 136–143. 10.1016/j.virol.2008.07.024 18752820

[B9] Chong K. T. Xiang L. Wang X. Jun E. L. Xi L. F. Schweinfurth J. M. (2006). High level expression of human epithelial beta-defensins (hBD-1, 2 and 3) in papillomavirus induced lesions. *Virol. J.* 3:75. 10.1186/1743-422x-3-75 16961924 PMC1579216

[B10] Cuperus T. Coorens M. van Dijk A. Haagsman H. P. (2013). Avian host defense peptides. *Dev. Comp. Immunol.* 41 352–369. 10.1016/j.dci.2013.04.019 23644014

[B11] Davis R. J. (1994). MAPKs: new JNK expands the group. *Trends Biochem. Sci.* 19 470–473. 10.1016/0968-0004(94)90132-5 7855889

[B12] de la Salle H. Altenburger W. Elkaim R. Dott K. Dieterle A. Drillien R. (1985). Active gamma-carboxylated human factor IX expressed using recombinant DNA techniques. *Nature* 316 268–270. 10.1038/316268a0 4040611

[B13] Derache C. Meudal H. Aucagne V. Mark K. J. Cadene M. Delmas A. F. (2012). Initial insights into structure-activity relationships of avian defensins. *J. Biol. Chem.* 287 7746–7755. 10.1074/jbc.M111.312108 22205704 PMC3293588

[B14] Ecco R. Brown C. Susta L. Cagle C. Cornax I. Pantin-Jackwood M. (2011). In vivo transcriptional cytokine responses and association with clinical and pathological outcomes in chickens infected with different Newcastle disease virus isolates using formalin-fixed paraffin-embedded samples. *Vet. Immunol. Immunopathol.* 141 221–229. 10.1016/j.vetimm.2011.03.002 21458080

[B15] Falco A. Chico V. Marroqui L. Perez L. Coll J. M. Estepa A. (2008). Expression and antiviral activity of a beta-defensin-like peptide identified in the rainbow trout (*Oncorhynchus mykiss*) EST sequences. *Mol. Immunol.* 45 757–765. 10.1016/j.molimm.2007.06.358 17692376

[B16] Frigo D. E. Tang Y. Beckman B. S. Scandurro A. B. Alam J. Burow M. E. (2004). Mechanism of AP-1-mediated gene expression by select organochlorines through the p38 MAPK pathway. *Carcinogenesis* 25 249–261. 10.1093/carcin/bgh009 14604893

[B17] Ganz T. (2003). Defensins: antimicrobial peptides of innate immunity. *Nat. Rev. Immunol.* 3 710–720. 10.1038/nri1180 12949495

[B18] Garcia-Valtanen P. Martinez-Lopez A. Ortega-Villaizan M. Perez L. Coll J. M. Estepa A. (2014). In addition to its antiviral and immunomodulatory properties, the zebrafish beta-defensin 2 (zfBD2) is a potent viral DNA vaccine molecular adjuvant. *Antiviral Res.* 101 136–147. 10.1016/j.antiviral.2013.11.009 24286781

[B19] Gong T. Jiang Y. Wang Y. Yang D. Li W. Zhang Q. (2010). Recombinant mouse beta-defensin 2 inhibits infection by influenza A virus by blocking its entry. *Arch. Virol.* 155 491–498. 10.1007/s00705-010-0608-1 20195655

[B20] Grubor B. Gallup J. M. Meyerholz D. K. Crouch E. C. Evans R. B. Brogden K. A. (2004). Enhanced surfactant protein and defensin mRNA levels and reduced viral replication during parainfluenza virus type 3 pneumonia in neonatal lambs. *Clin. Diagn. Lab. Immunol.* 11 599–607. 10.1128/cdli.11.3.599-607.2004 15138188 PMC404576

[B21] Guo H. Liu X. Xu Y. Han Z. Shao Y. Kong X. (2014). A comparative study of pigeons and chickens experimentally infected with PPMV-1 to determine antigenic relationships between PPMV-1 and NDV strains. *Vet. Microbiol.* 168 88–97. 10.1016/j.vetmic.2013.11.002 24314393

[B22] Han S. H. Kim Y.-E. Park J.-A. Park J.-B. Kim Y.-S. Lee Y. (2009). Expression of human β-defensin-2 gene induced by CpG-DNA in human B cells. *Biochem. Biophys. Res. Commun.* 389 443–448. 10.1016/j.bbrc.2009.08.162 19732743

[B23] Hazrati E. Galen B. Lu W. Wang W. Ouyang Y. Keller M. J. (2006). Human alpha- and beta-defensins block multiple steps in herpes simplex virus infection. *J. Immunol.* 177 8658–8666. 10.4049/jimmunol.177.12.8658 17142766

[B24] Hoover D. M. Wu Z. Tucker K. Lu W. Lubkowski J. (2003). Antimicrobial characterization of human β-defensin 3 derivatives. *Antimicrob. Agents Chemother.* 47 2804–2809. 10.1128/AAC.47.9.2804-2809.2003 12936977 PMC182640

[B25] Keestra A. M. de Zoete M. R. Bouwman L. I. van Putten J. P. (2010). Chicken TLR21 is an innate CpG DNA receptor distinct from mammalian TLR9. *J. Immunol.* 185 460–467. 10.4049/jimmunol.0901921 20498358

[B26] Klotman M. E. Chang T. L. (2006). Defensins in innate antiviral immunity. *Nat. Rev. Immunol.* 6 447–456. 10.1038/nri1860 16724099

[B27] Kogut M. H. Chiang H. I. Swaggerty C. L. Pevzner I. Y. Zhou H. (2012). Gene expression analysis of toll-like receptor pathways in heterophils from genetic chicken lines that differ in their susceptibility to *Salmonella* enteritidis. *Front. Genet.* 3:121. 10.3389/fgene.2012.00121 22783275 PMC3389315

[B28] Krisanaprakornkit S. Kimball J. R. Dale B. A. (2002). Regulation of human beta-defensin-2 in gingival epithelial cells: the involvement of mitogen-activated protein kinase pathways, but not the NF-kappaB transcription factor family. *J. Immunol.* 168 316–324. 10.4049/jimmunol.168.1.316 11751976

[B29] Kyriakis J. M. Avruch J. (2012). Mammalian MAPK signal transduction pathways activated by stress and inflammation: a 10-year update. *Physiol. Rev.* 92 689–737. 10.1152/physrev.00028.2011 22535895

[B30] Le Goffic R. Balloy V. Lagranderie M. Alexopoulou L. Escriou N. Flavell R. (2006). Detrimental contribution of the Toll-like receptor (TLR)3 to influenza A virus-induced acute pneumonia. *PLoS Pathog.* 2:e53. 10.1371/journal.ppat.0020053 16789835 PMC1475659

[B31] Lee H. Y. Takeshita T. Shimada J. Akopyan A. Woo J. I. Pan H. (2008). Induction of beta defensin 2 by NTHi requires TLR2 mediated MyD88 and IRAK-TRAF6-p38MAPK signaling pathway in human middle ear epithelial cells. *BMC Infect. Dis.* 8:87. 10.1186/1471-2334-8-87 18578886 PMC2447838

[B32] Lehrer R. I. Cole A. M. Selsted M. E. (2012). θ-Defensins: cyclic peptides with endless potential. *J. Biol. Chem.* 287 27014–27019. 10.1074/jbc.R112.346098 22700960 PMC3411038

[B33] Lehrer R. I. Daher K. Ganz T. Selsted M. E. (1985). Direct inactivation of viruses by MCP-1 and MCP-2, natural peptide antibiotics from rabbit leukocytes. *J. Virol.* 54 467–472. 2985808 10.1128/jvi.54.2.467-472.1985 PMC254818

[B34] Lehrer R. I. Ganz T. (2002). Defensins of vertebrate animals. *Curr. Opin. Immunol.* 14 96–102. 10.1016/S0952-7915(01)00303-X 11790538

[B35] Leikina E. Delanoe-Ayari H. Melikov K. Cho M. S. Chen A. Waring A. J. (2005). Carbohydrate-binding molecules inhibit viral fusion and entry by crosslinking membrane glycoproteins. *Nat. Immunol.* 6 995–1001. 10.1038/ni1248 16155572

[B36] Lewis S. B. Prior A. Ellis S. J. Cook V. Chan S. S. Gelson W. (2016). Flagellin Induces beta-Defensin 2 in Human Colonic Ex vivo Infection with Enterohemorrhagic *Escherichia coli*. *Front. Cell Infect. Microbiol.* 6:68. 10.3389/fcimb.2016.00068 27446815 PMC4914554

[B37] Li B. Ye J. Lin Y. Wang M. Zhu J. (2014). Preparation and identification of a single-chain variable fragment antibody against Newcastle diseases virus F48E9. *Vet. Immunol. Immunopathol.* 161 258–264. 10.1016/j.vetimm.2014.08.009 25183016

[B38] Li Y. Xu Q. Zhang T. Gao M. Wang Q. Han Z. (2015). Host avian beta-defensin and toll-like receptor responses of pigeons following infection with pigeon paramyxovirus type 1. *Appl. Environ. Microbiol.* 81 6415–6424. 10.1128/aem.01413-15 26162868 PMC4542233

[B39] Liu W. Q. Tian M. X. Wang Y. P. Zhao Y. Zou N. L. Zhao F. F. (2012). The different expression of immune-related cytokine genes in response to velogenic and lentogenic Newcastle disease viruses infection in chicken peripheral blood. *Mol. Biol. Rep.* 39 3611–3618. 10.1007/s11033-011-1135-1 21728003

[B40] Lynn D. J. Higgs R. Lloyd A. T. O’Farrelly C. Herve-Grepinet V. Nys Y. (2007). Avian beta-defensin nomenclature: a community proposed update. *Immunol. Lett.* 110 86–89. 10.1016/j.imlet.2007.03.007 17467809

[B41] Ma D. Lin L. Zhang K. Han Z. Shao Y. Liu X. (2011). Three novel *Anas platyrhynchos* avian beta-defensins, upregulated by duck hepatitis virus, with antibacterial and antiviral activities. *Mol. Immunol.* 49 84–96. 10.1016/j.molimm.2011.07.019 21856003

[B42] Ma D. Zhang K. Zhang M. Xin S. Liu X. Han Z. (2012). Identification, expression and activity analyses of five novel duck beta-defensins. *PLoS One* 7:e47743. 10.1371/journal.pone.0047743 23112840 PMC3480435

[B43] Mayo M. A. (2002). A summary of taxonomic changes recently approved by ICTV. *Arch. Virol.* 147 1655–1663. 10.1007/s007050200039 12181683

[B44] Motulsky. (2007). *GraphPad Prism(R) Version 5.0 Statistics Guide [M]*. San Diego, CA: GraphPad Software, Inc.

[B45] Pauwels R. Balzarini J. Baba M. Snoeck R. Schols D. Herdewijn P. (1988). Rapid and automated tetrazolium-based colorimetric assay for the detection of anti-HIV compounds. *J. Virol. Methods* 20 309–321. 10.1016/0166-0934(88)90134-6 2460479

[B46] Peroval M. Y. Boyd A. C. Young J. R. Smith A. L. (2013). A critical role for MAPK signalling pathways in the transcriptional regulation of toll like receptors. *PLoS One* 8:e51243. 10.1371/journal.pone.0051243 23405061 PMC3566169

[B47] Plotnikov A. Zehorai E. Procaccia S. Seger R. (2011). The MAPK cascades: signaling components, nuclear roles and mechanisms of nuclear translocation. *Biochim. Biophys. Acta* 1813 1619–1633. 10.1016/j.bbamcr.2010.12.012 21167873

[B48] Proud D. Sanders S. P. Wiehler S. (2004). Human rhinovirus infection induces airway epithelial cell production of human beta-defensin 2 both in vitro and in vivo. *J. Immunol.* 172 4637–4645. 10.4049/jimmunol.172.7.4637 15034083

[B49] Quinones-Mateu M. E. Lederman M. M. Feng Z. M. Chakraborty B. Weber J. Rangel H. R. (2003). Human epithelial beta-defensins 2 and 3 inhibit HIV-1 replication. *AIDS* 17 F39–F48. 10.1097/01.aids.0000096878.73209.4f 14571200

[B50] Ramasamy K. T. Reddy M. R. Verma P. C. Murugesan S. (2012). Expression analysis of turkey (*Meleagris gallopavo*) toll-like receptors and molecular characterization of avian specific TLR15. *Mol. Biol. Rep.* 39 8539–8549. 10.1007/s11033-012-1709-6 22699880

[B51] Rasoli M. Yeap S. K. Tan S. W. Moeini H. Ideris A. Bejo M. H. (2014). Alteration in lymphocyte responses, cytokine and chemokine profiles in chickens infected with genotype VII and VIII velogenic Newcastle disease virus. *Comp. Immunol. Microbiol. Infect. Dis.* 37 11–21. 10.1016/j.cimid.2013.10.003 24225159

[B52] Reed L. J. Muench H. (1938). A simple method of estimating fifty per cent endpoints. *Am. J. Epidemiol.* 27 493–497. 10.1093/oxfordjournals.aje.a118408

[B53] Rock F. L. Hardiman G. Timans J. C. Kastelein R. A. Bazan J. F. (1998). A family of human receptors structurally related to Drosophila Toll. *Proc. Natl. Acad. Sci. U.S.A.* 95 588–593. 10.1073/pnas.95.2.588 9435236 PMC18464

[B54] Roux P. P. Blenis J. (2004). ERK and p38 MAPK-activated protein kinases: a family of protein kinases with diverse biological functions. *Microbiol. Mol. Biol. Rev.* 68 320–344. 10.1128/mmbr.68.2.320-344.2004 15187187 PMC419926

[B55] Sano K. Ogawa H. (2014). Hemagglutination (inhibition) assay. *Methods Mol. Biol.* 1200 47–52. 10.1007/978-1-4939-1292-6_4 25117223

[B56] Sato S. Sanjo H. Takeda K. Ninomiya-Tsuji J. Yamamoto M. Kawai T. (2005). Essential function for the kinase TAK1 in innate and adaptive immune responses. *Nat. Immunol.* 6 1087–1095. 10.1038/ni1255 16186825

[B57] Seger R. Krebs E. G. (1995). The MAPK signaling cascade. *FASEB J.* 9 726–735. 10.1096/fasebj.9.9.7601337 7601337

[B58] Shahsavandi S. Ebrahimi M. M. Mohammadi A. Zarrin Lebas N. (2013). Impact of chicken-origin cells on adaptation of a low pathogenic influenza virus. *Cytotechnology* 65 419–424. 10.1007/s10616-012-9495-5 23011740 PMC3597177

[B59] Virella-Lowell I. Poirier A. Chesnut K. A. Brantly M. Flotte T. R. (2000). Inhibition of recombinant adeno-associated virus (rAAV) transduction by bronchial secretions from cystic fibrosis patients. *Gene Ther.* 7 1783–1789. 10.1038/sj.gt.3301268 11083501

[B60] Wang Y. Liu W. Tian M. Lin Y. Zhao F. Shi M. (2012). Downregulation of IL-15 and IL-18 in chicken embryo fibroblasts in response to virulent or lentogenic newcastle disease virus infection. *Inform. Technol. Agric. Eng.* 789–800. 10.1007/978-3-642-27537-1_93

[B61] Whitmarsh A. J. (2007). Regulation of gene transcription by mitogen-activated protein kinase signaling pathways. *Biochim. Biophys. Acta* 1773 1285–1298. 10.1016/j.bbamcr.2006.11.011 17196680

[B62] Xing Z. Cardona C. J. Anunciacion J. Adams S. Dao N. (2010). Roles of the ERK MAPK in the regulation of proinflammatory and apoptotic responses in chicken macrophages infected with H9N2 avian influenza virus. *J. Gen. Virol.* 91(Pt 2), 343–351. 10.1099/vir.0.015578-0 19864500

[B63] Xu Q. Chen Y. Zhao W. Zhang T. Liu C. Qi T. (2016). Infection of goose with genotype VIId Newcastle disease virus of goose origin elicits strong immune responses at early stage. *Front. Microbiol.* 7:1587. 10.3389/fmicb.2016.01587 27757109 PMC5047883

[B64] Xu Y. Zhang T. Xu Q. Han Z. Liang S. Shao Y. (2015). Differential modulation of avian beta-defensin and Toll-like receptor expression in chickens infected with infectious bronchitis virus. *Appl. Microbiol. Biotechnol.* 99 9011–9024. 10.1007/s00253-015-6786-8 26142390 PMC7080159

[B65] Yamamoto M. Takeda K. (2010). Current views of toll-like receptor signaling pathways. *Gastroenterol. Res. Pract.* 2010:240365. 10.1155/2010/240365 21197425 PMC3010626

[B66] Yang D. Biragyn A. Hoover D. M. Lubkowski J. Oppenheim J. J. (2004). Multiple roles of antimicrobial defensins, cathelicidins, and eosinophil-derived neurotoxin in host defense. *Annu. Rev. Immunol.* 22 181–215. 10.1146/annurev.immunol.22.012703.104603 15032578

[B67] Yang D. Chertov O. Bykovskaia S. N. Chen Q. Buffo M. J. Shogan J. (1999). β-defensins: linking innate and adaptive immunity through dendritic and T cell CCR6. *Science* 286 525–528. 10.1126/science.286.5439.525 10521347

[B68] Yasin B. Wang W. Pang M. Cheshenko N. Hong T. Waring A. J. (2004). Theta defensins protect cells from infection by herpes simplex virus by inhibiting viral adhesion and entry. *J. Virol.* 78 5147–5156. 10.1128/JVI.78.10.5147-5156.2004 15113897 PMC400355

[B69] Yoneyama M. Kikuchi M. Natsukawa T. Shinobu N. Imaizumi T. Miyagishi M. (2004). The RNA helicase RIG-I has an essential function in double-stranded RNA-induced innate antiviral responses. *Nat. Immunol.* 5 730–737. 10.1038/ni1087 15208624

[B70] Zhao H. Zhou J. Zhang K. Chu H. Liu D. Poon V. K. (2016). A novel peptide with potent and broad-spectrum antiviral activities against multiple respiratory viruses. *Sci. Rep.* 6:22008. 10.1038/srep22008 26911565 PMC4766503

